# Community Disassembly in a Fragmented Tropical Landscape Driven by Both Deterministic and Stochastic Processes

**DOI:** 10.1002/ece3.72687

**Published:** 2026-01-09

**Authors:** Isham Azhar, Hendra F. Sihaloho, Matthew J. Struebig, Juliana Senawi, Stephen J. Rossiter, Caleb D. Phillips, Tigga Kingston

**Affiliations:** ^1^ Department of Biological Sciences Texas Tech University Lubbock Texas USA; ^2^ Durrell Institute of Conservation and Ecology University of Kent Canterbury UK; ^3^ University Kebangsaan Malaysia Bangi Malaysia; ^4^ School of Biological and Behavioural Sciences Queen Mary University of London London UK; ^5^ Department of Evolution, Ecology, and Behavior University of Illinois Urbana‐Champaign Urbana Illinois USA

**Keywords:** community assembly, community disassembly, conservation, fragmentation, functional diversity, niche

## Abstract

Deforestation is a key driver of habitat loss, transforming extensive forested areas into fragmented, isolated patches with reduced biodiversity. While the patterns of species loss from fragmentation are well documented, the underlying processes driving these patterns remain unclear. We sought to identify the community processes driving the disassembly of tropical insectivorous bat communities in response to forest fragmentation in Malaysia. We measured species richness and four functional diversity metrics across assemblages in continuous forests and forest fragments of varying sizes. Eight traits related to prey detection, acquisition, and processing were used to characterize functional diversity based on a global pool of captured species. We found that species‐poor assemblages represented nested subsets of species‐rich assemblages, indicating that species loss is non‐random. This non‐random loss led to a collapse of functional trait space between 11 and 8 species before stabilizing at a lower richness. Analyses of functional diversity against null expectations showed that assemblages in continuous forests were structured by environmental filtering and niche packing, whereas persistence in fragments was driven by stochastic processes. This pattern, alongside the random occupation of fragments, suggests that fragmentation‐driven disassembly likely arises from a complex interplay between deterministic and stochastic processes. Insights regarding the relative roles of determinism and stochasticity presented herein highlight the collective contribution of habitat fragments to overall landscape‐level diversity and underscore the challenges in identifying priority fragments for conservation. They also emphasize the importance of incorporating functional diversity, rather than solely fragment size and species counts, in landscape‐level conservation planning.

## Graphical abstract

This study investigates the mechanisms driving community disassembly in a fragmented landscape using a functional diversity framework. Forest fragmentation drives non‐random species loss among forest‐interior insectivorous bat assemblages across the landscape. This leads to a contraction of functional trait space as species richness declines from 11 to 8 and levels off at a reduced level. Disassembly appears to result from the interplay of both deterministic and stochastic processes. These findings highlight the importance of incorporating trait‐based approaches into conservation planning for fragmented landscapes and the challenges involved in identifying priority fragments for conservation.

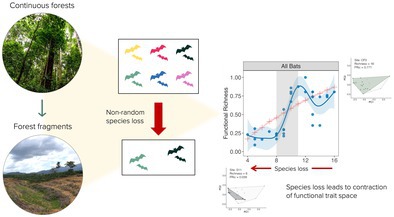

## 1 Introduction

Habitat fragmentation is widely recognized as a driver of biodiversity loss, reducing the quantity, quality, and connectivity of habitats available to species in disturbed landscapes (Haddad et al. 2015; Kuipers et al. 2021). However, recent studies have indicated that fragmentation may have varied outcomes for species richness when considered independently from habitat loss, ranging from negative or negligible to positive (Fahrig 2017; Rybicki et al. 2020).

Traditionally, the impacts of habitat fragmentation on biological communities have been assessed using measures of species diversity (Fahrig 2017). While this approach effectively reveals the patterns that result from fragmentation, it provides a limited explanation of the underlying processes driving community disassembly in fragmented landscapes. Understanding these community processes can also help predict management outcomes across different habitats and communities (Funk et al. 2023). Trait‐based approaches provide a means to accomplish this and use organismal traits that consider interactions within and between species and with their environment, thereby allowing the measurement of functional diversity (Mouchet et al. 2010).

Various measures of functional diversity have been developed to capture different components of the functional space indicative of interspecific and environmental processes structuring assemblages (Villéger et al. 2008; Laliberté and Legendre 2010; Mouillot et al. 2013). These metrics can be used to describe shifts in the functional space of biological communities in response to anthropogenic perturbations (e.g., Hooper et al. 2000; Villéger et al. 2008). Deviations of the observed metrics from null expectations are then evaluated to infer the contribution of deterministic (e.g., environmental filtering, limiting similarity) or stochastic (e.g., ecological drift, dispersal) processes (e.g., Ortega‐Martínez et al. 2020; Chakravarty et al. 2021). Although several studies have found a general decline in functional diversity associated with fragmentation, suggesting loss of species with particular traits or trait combinations (e.g., Girão et al. 2007; Zambrano et al. 2019), there has been little consideration of the community processes governing assemblage structure in fragmented landscapes.

Insectivorous bats are a key component of paleotropical forest diversity, with assemblages in unmodified forests exceeding 60 species (Kingston et al. 2003). However, many species, particularly those with acoustic and flight morphologies that optimize foraging for insects in the structurally complex vegetation of the forest interior, are proving susceptible to forest loss and fragmentation (Struebig et al. 2011; Kingston 2013; Huang et al. 2019). Within this vulnerable forest interior ensemble, species with low vagility that roost in forest structures, such as standing and dead tree hollows or leaves, exhibit greater sensitivity to disturbance than the more vagile cave‐roosting species (Struebig et al. 2011; Rossiter et al. 2012; Huang et al. 2019). Although all species are insectivorous, they exhibit significant morphological diversity, particularly in traits associated with prey detection, acquisition, and processing. This trait variation likely drives resource partitioning among species, permitting coexistence in unmodified habitats (Kingston et al. 2000; Schmieder et al. 2012; Senawi et al. 2015; Senawi and Kingston 2019). Moreover, variations in traits such as echolocation and wing morphology are associated with species persistence and vulnerability following disturbances (Kingston 2013; Huang et al. 2019).

Although it has been established that particular traits render certain paleotropical bat species more vulnerable to disturbances, here we sought to determine the role of community processes, specifically environmental filtering and competition, in species persistence and loss in a fragmented landscape. Our objective was to identify the processes underlying community disassembly for forest interior insectivorous bats in a severely fragmented landscape in Malaysia, using a functional diversity framework. To identify the community processes leading to disassembly following fragmentation, we estimated the deviation of functional diversity metrics from null expectations along a fragmentation gradient and across a large tract of continuous forest. We hypothesized that if environmental filtering was the primary process driving disassembly, we would see a contraction of the overall functional trait space across the gradient. In contrast, limiting similarity indicative of competition would be shown by increased distances and regularity between neighbors in functional space.

## 2 Materials and Methods

### 2.1 Experimental Design

Insectivorous bats were sampled from 26 forest fragments of different sizes (small—mean 70 ha, range 31–102 ha, medium—mean 353 ha, range 251–433 ha, and large—mean 5410 ha, range 2025–11,339 ha). The fragments varied in isolation from the nearest forest fragments (0.6–2.3 km) and distances to the nearest continuous forest (2.1–11.0 km). Additionally, bat sampling was conducted at six sites within the continuous forest of the Tengku Hasanal Wildlife Reserve (formerly Krau Wildlife Reserve; Struebig et al. 2008). Most of the bat capture data were sourced from Struebig et al. (2008). In addition, data on bat captures from 2019 to 2022 were obtained from two sites within the continuous forests situated within Tengku Hasanal Wildlife Reserve, namely S01 and S06. See Supporting Information S1 for more details on bat sampling.

### 2.2 Species Trait Data

To generate functional diversity metrics, we selected eight traits that relate to a species' ability to fly in complex vegetation, and to detect, capture, and handle its insect prey: (a) body mass (g), (b) forearm length (mm), (c) wing area (m^2^), (d) wingspan (m), (e) maximum bite force (N), (f) echolocation call duration (ms), (g) echolocation call start frequency (kHz), and (h) echolocation call bandwidth (kHz) (Table S1).

### 2.3 Fragmentation Measures

The effect of fragmentation on measures of functional diversity was tested using three widely used fragmentation measures: fragment area (ha) (hereafter “area”), the shortest Euclidean distance to the nearest unmodified forests (km) (hereafter “isolation”), and distance to the nearest fragment (km) (hereafter “nearest fragment”) (Watling and Donnelly 2006). Multicollinearity among fragmentation measures was assessed using Pearson's correlation. Pairwise correlations were low (*r* < 0.3, *p* > 0.05), indicating that the measures were independent of each other. All three measures were logarithmically transformed to approximate normal distributions (Table S2).

### 2.4 Community Matrix

We used a rarefied abundance‐based approach based on Hill numbers (*q* = 2) to establish the 93% sampling coverage estimates for all sites. This threshold allowed extrapolation up to approximately twice the reference sample size, even in sites with low species richness and abundance (Hsieh et al. 2016). Using the reference sample size determined for 93% coverage, we generated a community matrix that included all bat species. This matrix was created by resampling species based on their occurrence probabilities derived from observed abundances at each site. Since resampling was weighted by observed abundances, common species were more likely to be selected than rare species. Consequently, two low‐abundance species were not included in the resampled communities, consistent with the probabilistic nature of the method. Importantly, preliminary analyses indicated that their exclusion did not meaningfully influence subsequent functional diversity results. The excluded species were not among those with extreme trait values that occupy the periphery of the trait space. Instead, the overall trait space was largely shaped by more common forest‐roosting species and a cave‐roosting species known to be one of the largest in the study system.

Due to the differential response to habitat disturbance, which is likely underpinned by differences in trait combinations (Kingston 2013), we created separate matrices for cave‐roosting and forest‐roosting bat species for each site. Forest‐roosting species primarily utilize tree hollows, foliage, or similar structures. In contrast, cave‐roosting species roost in caves and rely on forests mainly for foraging. Species were classified as cave‐roosting or forest‐roosting based on our prior work in the system (Kingston et al. 2006; Struebig et al. 2008; Kingston 2013). Although some species occasionally roost outside their primary category, there is no evidence of systematic roost switching in our study system. Accordingly, the primary roosting strategy was used as the relevant ecological distinction. Further details on the construction of the community matrices can be found in the Supporting Information S1.

### 2.5 Assessing the Functional Structure of Bat Assemblages

We used Principal Component Analysis (PCA) to construct a multidimensional functional trait space based on species trait‐based distance. This functional trait space was then used to calculate four functional diversity metrics: functional richness (hereafter, FRic), functional dispersion (hereafter, FDis), mean nearest‐neighbor distance (hereafter, FNND), and functional identity (hereafter, FIde) (Table 1). These metrics are among the most suitable measures for assessing assembly mechanisms (Villéger et al. 2008; Mouillot et al. 2013). We added FIde to identify which traits most influenced the persistence or loss of species through the disassembly process. We repeated this procedure using community matrices for each site, retaining only cave‐roosting and forest‐roosting bats since these bats have different susceptibilities to fragmentation. Further details on the functional diversity metrics are provided in the Supporting Information S1.

**TABLE 1:** Summary of the functional diversity metrics used in this study and expected outcomes and processes through disassembly.

Functional diversity metric	Acronym	Definition	Process interpretations in this study
Functional richness (Villéger et al. 2008)	FRic	The proportion of functional trait space occupied by an assemblage relative to the pooled assemblage	Environmental filtering typically reduces FRic
Functional dispersion (Laliberté and Legendre 2010)	FDis	The abundance‐weighted mean distance of all taxa from the center of the functional space	Environmental filtering results in lower FDis because species' traits are less spread out from the centroid. Meanwhile, competition leads to an increase in the spread of trait values from the centroid
Functional mean nearest neighbor distance (Weiher et al. 1998)	FNND	The mean of weighted distances to the nearest neighbor within the functional trait space of an assemblage	Environmental filtering leads to lower FNND because species with similar functional traits are clustered together. In contrast, competition increases FNND due to greater dissimilarity in traits among nearest neighbors within a community
Functional identity (Mouillot et al. 2013)	FIde	The average position of species along each axis of the functional trait space, determined by the mean trait values weighted by abundance	Shifts in mean trait values towards traits that confer adaptation to environmental conditions or competitive advantage can help distinguish between the effects of environmental filtering and competition

### 2.6 Null Models

To evaluate how species loss affects the overall size of functional trait space and density within the space, we conducted randomization tests comparing the observed values of FRic and FNND to those derived from a null distribution. Specifically, we employed a richness‐constrained null model that preserves the number of species at each site while randomizing species assignment from the regional pool (Gotelli 2000). Observed and null expected values of FRic and FNND were calculated at each species richness value for the full community matrix that included all bat species, as well as for submatrices considering solely cave‐roosting and forest‐roosting bats.

To identify the community processes driving disassembly in response to fragmentation, we calculated standardized effect sizes (SES) for each functional diversity metric. These SES values were derived by comparing the observed values with those expected under a null distribution using the “independentswap” method. This method preserves both the number of species at each site and the overall frequency of each species across sites while randomizing species co‐occurrence (Gotelli 2000). The null model was applied to both the full community matrix and the submatrices. The 95% confidence interval for each of the SES values was calculated. SES with a confidence limit greater or less than zero signifies that a particular metric is significantly higher or lower than expected by the null model. Further details on the null models are provided in the Supporting Information S1.

### 2.7 Statistical Analyses

We used General Additive Modeling (GAM) to assess how the overall functional trait space, as well as the packing of the trait space, is affected by species loss. Generalized linear models (GLMs) were used to test the influence of each of the fragmentation measures on species richness and each of the functional diversity metrics for the full matrix and submatrices. The Akaike information criterion corrected for small samples (AICc) was used to determine the most plausible model.

We performed a nestedness analysis to determine the extent to which the assemblages exhibited nested patterns when fragments were ordered by species richness. The order of forest fragments in the maximally nested matrix was correlated with forest fragmentation measures to assess whether the maximally nested matrices produced an ecologically meaningful nested arrangement relative to forest fragmentation. Separate analyses were conducted for all bat species in the study, as well as submatrices specifically for cave‐roosting and forest‐roosting bats. All analyses were conducted in R version 2023.06.0 + 421 (R Core Team 2023). See Supporting Information S1 for more details on the nestedness analysis.

## 3 Results

### 3.1 Nestedness of Assemblages

Nestedness analyses were performed to determine if the patterns of species loss in forest fragments in response to habitat fragmentation were non‐random with respect to forest fragmentation. Assemblages of all bats were significantly nested when ordered by species richness (*p* < 0.05). The maximally nested arrangement was positively correlated with isolation (km) (Spearman's correlation, *p* = 0.015) and weakly correlated with area (ha) (Spearman's correlation, *p* = 0.05), but not with the distance to the nearest fragment (km). For forest‐roosting bats, although assemblages were not significantly nested when ordered by richness, there was a correlation between richness order and both area (Spearman's correlation, *p* < 0.026) and isolation (Spearman's correlation, *p* = 0.01), but not with the distance to the nearest fragment. In contrast, there was no nested pattern or correlation between the nested order of sites and fragmentation for cave‐roosting bats.

### 3.2 Functional Trait Space Structure Shifts With Declining Species Richness

**FIGURE 1:**
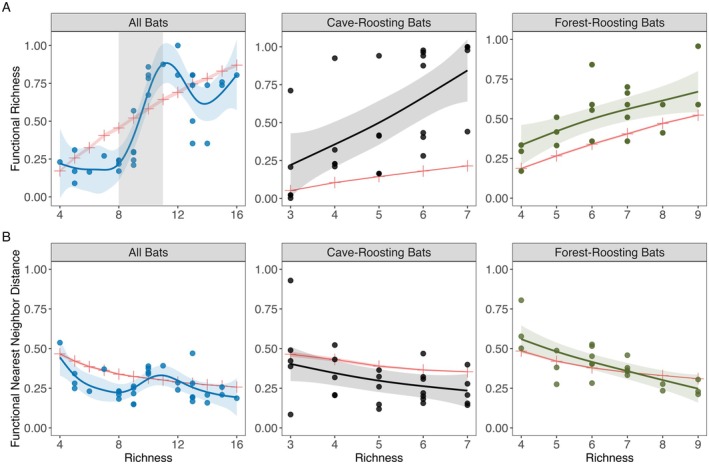
Contraction of functional trait space (FRic) and decrease in trait packing (FNND) in response to decreasing species richness and comparison with null expectations. Panels show the relationship between species richness and (A) observed functional richness (FRic), and (B) observed mean nearest neighbor distance (FNND) for all bats, cave‐roosting bats, and forest‐roosting bats. Shaded region represents 95% confidence intervals for the observed FRic and observed FNND. Red lines represent FRic and FNND values at each richness level under the null model. Shaded box in (A) highlights the phase of functional trait space contraction.

We used generalized additive modeling (GAM) to examine the influence of declines in species richness on observed FRic, FNND, and species richness. The observed relationships of FRic and FNND with species richness were compared to the responses predicted under null expectations. We found FRic decreased with decreasing species richness for all bats (*p* < 0.0001), cave‐roosting bats (*p* < 0.001), and forest‐roosting bats (*p* < 0.01) (Figure 1A, Table 2A), suggesting that the functional trait space contracted as species richness decreased.

**TABLE 2:** Detailed summary of Generalized Additive Model (GAM) results examining the relationship between species richness and the observed functional richness (FRic) and functional mean nearest neighbor distance (FNND).

Functional richness (FRic)				
Parametric coefficients				
	Estimate	Standard error	*t*‐vaIue	Pr(>|*z*|)
All bats	0.504	0.024	20.82	< 0.0001
Cave‐roosting bats	0.529	0.055	9.682	< 0.0001
Forest‐roosting bats	0.514	0.032	16.14	< 0.0001

Approximate significance of smooth terms				
	Effective degrees of freedom (edf)	Reference degrees of freedom (Ref.df)	*F*‐value	*p*‐value
*S(Richness)*				
All bats	6.252	7.435	13.88	< 0.0001
Cave‐roosting bats	1	1	18.56	< 0.001
Forest‐roosting bats	1	1	12.25	0.002

Functional mean nearest neighbor distance (FNND)				
Parametric coefficients				
	Estimate	Standard error	*t*‐value	Pr(>|*z*|)
All bats	0.269	0.012	22.42	< 0.0001
Cave‐roosting bats	0.306	0.031	9.877	< 0.0001
Forest‐roosting bats	0.398	0.021	19.41	< 0.0001

Approximate significance of smooth terms				
	Effective degrees of freedom (edf)	Reference degrees of freedom (Ref.df)	*F*‐value	*p*‐value
*S(Richness)*				
All bats	7.588	8.509	3.938	< 0.01
Cave‐roosting bats	1.133	1.254	4.811	0.04
Forest‐roosting bats	1	1	23.57	< 0.0001

FRic for all bats exhibited a two‐phase relationship with richness. Functional trait space contracted when species richness was between 11 and 8 species but remained largely unchanged at lower richness. This pattern contrasts with the null expectations, which predict a steady decline in functional trait space as species richness decreases (Figure 1A). In contrast, the responses for cave‐roosting and forest‐roosting bats followed the null model predictions of a linear decline. However, in both cases, FRic was greater than expected at any given level of richness, particularly for cave‐roosting bats. They also exhibited a greater reduction in functional trait space than null expectations compared to the forest‐roosting bats (Figure 1A).

We found that FNND increased with decreasing species richness for all bats (*p* < 0.01), cave‐roosting bats (*p* < 0.05), and forest‐roosting bats (*p* < 0.0001) (Figure 1B, Table 2B), indicating that the functional trait space became less densely packed as species richness decreased. Additionally, the responses of FNND to decreasing species richness for all bats, cave‐roosting bats, and forest‐roosting bats aligned with null expectations, showing a continuous increase in FNND as species richness decreased (Figure 1B).

### 3.3 Effects of Fragmentation on Functional Diversity

We examined the effects of fragmentation on species richness and the observed functional diversity metrics using generalized linear models (GLM). FRic was positively associated with fragment area (*p* = 0.015) and negatively associated with isolation (*p* = 0.01) for all bats (Table 3A). Furthermore, there was a negative association between FNND and isolation (*p* = 0.037) (Table 3A). For cave‐roosting bats, the best model indicated that FRic decreases with the nearest fragment (*p* = 0.027) and isolation (*p* = 0.03) (Table 3B). In contrast, for forest‐roosting bats, FRic was positively associated with the nearest fragment (*p* = 0.038) (Table 3C). The full performance of the GLMs is detailed in Tables S6–S8.

**TABLE 3:** Regression relationships between fragmentation measures and species richness and functional diversity metrics for all bats, cave‐roosting bats, and forest‐roosting bats, analyzed using generalized linear modeling (GLM).

Response variable		Predictor variables	Estimate	Standard error	*z*/*t*‐value	Pr(>|*z*|)
A.	All bats					
	Species richness	Area	0.078	0.051	1.435	0.151
	Functional richness	**Area**	**0.082**	**0.031**	**2.617**	**0.015**
		**Isolation**	**−0.213**	**0.076**	**−2.802**	**0.01**
	Functional dispersion	Isolation	−0.03	0.044	−0.666	0.512
	Functional mean nearest neighbor distance	**Isolation**	**−0.059**	**0.028**	**−2.143**	**0.043**
B.	Cave‐roosting bats					
	Species richness	Area	0.077	0.078	0.977	0.329
	Functional richness	Area	0.107	0.055	1.953	0.068
		**Nearest Fragment**	**−0.288**	**0.119**	**−2.425**	**0.027**
		**Isolation**	**−0.333**	**0.141**	**−2.366**	**0.03**
	Functional dispersion	Nearest Fragment	−0.095	0.145	0.653	0.522
	Functional mean nearest neighbor distance	Isolation	−0.341	0.193	−1.766	0.093
C.	Forest‐roosting bats					
	Species richness	Nearest Fragment	0.104	0.164	−0.635	0.526
	Functional richness	**Nearest Fragment**	**0.164**	**0.072**	**2.268**	**0.038**
	Functional dispersion	Nearest Fragment	0.043	0.022	1.965	0.068
	Functional mean nearest neighbor distance	Area	−0.078	0.05	−1.543	0.144

*Note:* *z*‐values are presented for species richness. Outputs of the final models selected through backward stepwise selection using the AICc criterion. Predictor variables with statistically significant *p*‐values are highlighted in bold. The intercept corresponds to log‐transformed predictor variables: A. Area—log of fragment area (ha), B. Nearest Fragment—log of distance to the nearest forest fragment (km), and C. Isolation—log of shortest Euclidean distance to the continuous forest (km).

### 3.4 Deterministic Versus Stochastic Processes Driving Assemblage Structure

**FIGURE 2:**
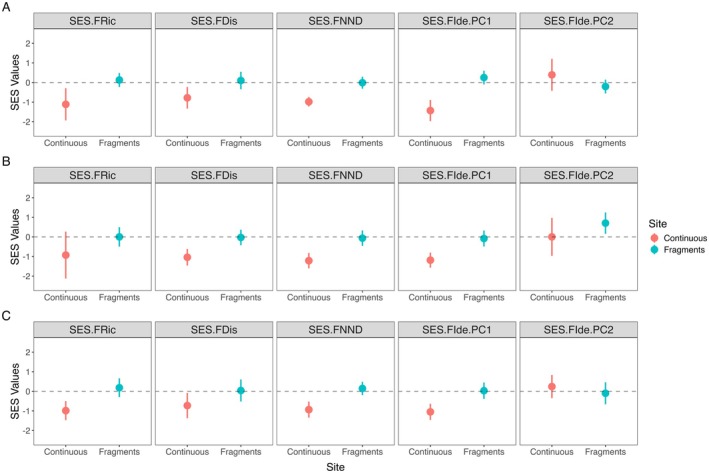
Deterministic processes primarily drive the structuring of assemblages in continuous forests, while stochastic processes structure assemblages in fragments. Standardized effect sizes for functional richness (SES.FRic), functional dispersion (SES.FDis), mean nearest neighbor distance (SES.FNND), and the functional identity of PC1 (SES.FIde.PC1) and PC2 (SES.FIde.PC2) are compared to null expectations. Panels display (A) all bats, (B) cave‐roosting bats, and (C) forest‐roosting bats. Filled circles indicate means, the whiskers represent 95% confidence intervals, and dashed horizontal lines at 0 represent null expectations. SES values below the 95% interval of the null indicate underdispersion (SES < 0), while values above indicate overdispersion (SES > 0). Site categories are Continuous—continuous forests, Fragments—forest fragments.

To determine the assembly and disassembly mechanisms driving changes in the overall functional trait space in response to fragmentation, we calculated the SES values for each functional metric for sites from continuous forests and fragments. Departures from null expectations were only detected in the continuous forest sites. Continuous forest assemblages of all bats exhibited underdispersion in SES values for FRic, FDis, FNND, and FIde PC1 (Figure 2A). For cave‐roosting bats, we found underdispersion in SES FDis, FNND, and FIde PC1 in the continuous forest sites, and overdispersion in the FIde PC2 in the fragments (Figure 2B). Whereas for forest‐roosting bats, we found underdispersion of SES FRic, FDis, FNND, and FIde PC1. None of the SES values in the fragments differed from null expectations. Our results suggest assemblages in the continuous forests are structured deterministically, whereas those in the fragments are structured by stochastic processes.

## 4 Discussion

The present study was designed to elucidate the processes governing the disassembly of insectivorous bat assemblages in an extensively fragmented landscape in peninsular Malaysia. Our models showed that changes in aspects of functional diversity were influenced by fragment area and isolation from the nearest continuous forest and other forest fragments. We observed that FRic decreased while FNND increased as species richness declined across the landscape. This pattern suggests that species loss results in a contraction and reduced density within the overall functional trait space in the study system. Additionally, our results from the null model analyses suggest the role of deterministic processes in structuring assemblages in the continuous forest, whereas the disassembly of assemblages in the fragments was driven by stochastic processes. We also observed that species‐poor assemblages were subsets of the species‐rich assemblages for the full community matrix, and the nested patterns were driven by isolation from the nearest continuous forests.

Our results indicate that fragmentation significantly impacts functional diversity, particularly FRic for all bat species, cave‐roosting and forest‐roosting bats, and FNND for all bat species. The observed parallel decline in FRic and the increase in FNND as species richness decreases suggest that species loss alters the functional trait space. We identified a threshold at the approximate mid‐point of species richness decline (11–8 species), at which the functional trait space rapidly contracts and then levels off at a reduced value. This non‐linear pattern likely reflects differences in species contributions to the functional trait space. The rapid contraction observed around the threshold appears to be driven by the loss of functionally distinctive species at the periphery of the trait space (Figure S1). These species often possess extreme trait values and disproportionately contribute to the expansion of the overall space as richness increases (Villéger et al. 2008; Mouchet et al. 2010). In our system, the non‐random loss of peripheral species leads to a contraction of functional trait space, reflecting a focus on species loss rather than on the trait space expansion with increasing richness, as reported in other systems. Beyond this threshold, further contraction may be limited since the loss of functionally similar species clustered near the center has little effect on the overall trait space, potentially reflecting functional redundancy among these species.

Similar patterns have been reported in other systems examining how richness influences the occupied functional trait space. Functional diversity saturates rapidly with increasing richness, and elevated functional redundancy beyond a certain threshold limits further expansion of trait space (e.g., fishes, Guillemot et al. 2011; forests, Monge‐Gonzáles et al. 2021). The contraction in our study may also reflect the sensitivity of FRic to species richness (Villéger et al. 2008; Mouchet et al. 2010), which is consistent with evidence that functional diversity is most informative when differences in alpha diversity are small (Poos et al. 2009). This relationship is particularly pronounced when grouping cave‐roosting and forest‐roosting bats, as phylogenetic similarity increases trait redundancy (Ng et al. 2022), potentially contributing to a non‐continuous collapse of functional trait space.

Based on the null model analyses, the underdispersion of FNND in the continuous forests indicates an increased functional trait space density, a pattern consistent with niche packing. The increased packing of functional trait space corroborates results from other taxa, which have revealed denser packing of niche space in regions of high productivity, particularly in areas of low elevations and latitudes (e.g., birds, Pigot et al. 2016; Pellisier et al. 2018). Additionally, the underdispersion of FRic and FDis suggests that environmental filtering shapes assemblage composition in continuous forests. Examination of the functional trait space reveals a reduced representation of species at the extremes of PC1 (body size). This may be influenced by our study design, as all harp traps were set across trails at ground level, where they are more effective at capturing smaller bats in the forest understorey (Francis 1989). Larger bats occasionally use large forest trails, but they typically perch or forage in higher forest strata, above the height of the traps (TK pers. obs.). Performance studies further suggest that larger bats are less able to negotiate the densely cluttered environment of the forest (Senawi and Kingston 2019). We therefore infer that the under‐representation of larger bats in the continuous forests may reflect an artifact of the trapping protocol rather than a true absence. Both environmental filtering and niche packing describe assemblages dominated by numerous similar species that are well adapted to local conditions (Kraft et al. 2015). These findings align with previous research highlighting specialized adaptations within the Paleotropical forest interior bat ensemble for navigating cluttered environments (Kingston et al. 2003; Kingston 2013).

The absence of significant deviations of functional diversity metrics from null expectations in the fragments suggests that disassembly is driven by stochastic processes. While non‐random species loss leads to a contraction of functional trait space, species persistence within individual fragments results in seemingly random occupancy. This paradox may reflect fine‐scale processes influencing species persistence and loss, shaping the contrasting occupancy patterns observed among the fragments. In this study, we assumed that the fragmentation measures used are important predictors of functional diversity. This implicit assumption is based on the expectation that vegetation structure and microclimate are comparable across fragments, which is reasonable given that all fragments were once part of the same continuous forest. However, even in relatively intact forests such as Tengku Hasanal Wildlife Reserve, small‐scale (< 1 km^2^) variations in topography and hydrology can shape species occurrence, resulting in compositional differences that may simply reflect sampling effects (TK, pers. obs.). These fine‐scale variations could partly explain the stochastic occupancy patterns we observed.

While fine‐scale natural heterogeneity may explain part of this pattern, anthropogenic variation adds another layer of complexity. Within the Krau landscape, varied land‐use activities may impose distinct pressures on individual fragments. These pressures can alter vegetation structure, thereby influencing both microclimatic stability (Terschanski et al. 2024) and resource availability (Fang et al. 2019). Such differences potentially exert localized deterministic filtering within individual fragments, producing occupancy patterns that appear random at landscape scales. Evidence from other systems indicates that fine‐scale deterministic filters can structure communities even when large‐scale patterns seem stochastic (Zhang et al. 2025). This parallel raises the possibility that a similar interplay may underlie the patterns we observed. Spatial and ecological variation may allow species reliant on specific resources to persist, while stochastic dispersal and fluctuations in abundance further reinforce the apparent randomness observed (Chase 2003). Collectively, these factors may decelerate species loss, although extinction debts likely persist (Halley and Pimm 2023). Nonetheless, the absence of significant differences from null expectations could be attributed to niche separation along dimensions not captured by our trait space (Pigot et al. 2016). Incorporating finer‐scale data on species interactions, as well as environmental and spatial variables, could reveal additional dimensions of community structure. Building on our findings, this approach would provide complementary insights into how fragmentation affects community composition and ecosystem functioning, revealing patterns not apparent at the coarse scale.

Our study shows that functional diversity can help reveal processes driving disassembly at the landscape level. In continuous forests, assembly is shaped by environmental filtering and niche packing, whereas the disassembly in the fragments is largely driven by stochastic processes. Although non‐random species loss following a nested subset pattern contracts the functional trait space, local stochasticity makes persistence within fragments less predictable. Consequently, relying on species richness and identity alone provides limited power to predict which fragments should be prioritized in similar landscapes. Fragment characteristics and connectivity, which often correlate with species richness, remain useful for prioritization (Marchesan and Kolasa 2024), but they overlook key aspects of functional composition. Integrating functional traits alongside taxonomic information can therefore strengthen management decisions (Meerback and Haesen 2025). Ground‐based assessments are also important since fine‐scale variability influencing species persistence may not be detected without field observations. Preserving functional diversity in fragmented systems requires managing fragments collectively rather than individually, emphasizing the collective role of fragments in maintaining functional diversity at the landscape scale. These findings highlight that functional traits and community assembly processes are essential considerations in conservation planning, beyond just patch sizes and species counts.

## Author Contributions

**Isham Azhar:** conceptualization (lead), data curation (lead), formal analysis (lead), investigation (lead), methodology (lead), project administration (lead), validation (lead), visualization (lead), writing – original draft (lead), writing – review and editing (lead). **Hendra F. Sihaloho:** data curation (equal), formal analysis (supporting), investigation (equal), methodology (supporting), writing – review and editing (equal). **Matthew J. Struebig:** data curation (equal), formal analysis (supporting), methodology (equal), validation (equal), writing – review and editing (equal). **Stephen J. Rossiter:** data curation (supporting), methodology (equal), validation (equal), writing – review and editing (equal). **Juliana Senawi:** investigation (supporting), methodology (supporting), project administration (supporting), writing – review and editing (supporting). **Caleb D. Phillips:** conceptualization (equal), data curation (equal), formal analysis (supporting), funding acquisition (lead), investigation (equal), methodology (equal), project administration (equal), supervision (lead), validation (equal), visualization (equal), writing – review and editing (equal). **Tigga Kingston:** conceptualization (equal), data curation (equal), formal analysis (supporting), funding acquisition (lead), investigation (equal), methodology (equal), project administration (equal), supervision (lead), validation (equal), visualization (equal), writing – original draft (supporting), writing – review and editing (equal).

## Disclosure

*Statement of inclusion*: This study brought together authors from diverse backgrounds and multiple countries, including scientists and local community members from the region where the research was conducted. The study benefited from a diverse range of perspectives and intellectual input on study design and research approach. Through capacity‐building efforts, Malaysian undergraduates were trained in various aspects of bat research and ecology. Many of these trainees have since advanced to professional roles within the country's industry or are pursuing advanced degrees, including master's and PhD programs.

## Conflicts of Interest

The authors declare no conflicts of interest.

## Supporting information




**Data S1:** Supporting Information.

**Data S2:** Supporting Information.

**Data S3:**Supporting Information.

## Data Availability

Data used to produce this study is uploaded as Supporting Information.
