# Supplementary material for: Community Disassembly in a Fragmented Tropical Landscape Driven by Both Deterministic and Stochastic Processes
Source: Ecol Evol. 2026 Jan 9;16(1):e72687. doi: 10.1002/ece3.72687 (PMC12789661; doi:10.1002/ece3.72687)
Supplement: Supplementary file 3 — Data S3:Supporting Information. [file ECE3-16-e72687-s001.docx]

Supporting Information

Study Site

The study was carried out in the Krau landscape in central Peninsular Malaysia. Historically, the landscape was covered with dipterocarp rainforest. However, industrial logging and land conversion for non-timber plantations, primarily oil palm (*Elaeis guineensis*) and rubber (*Hevea brasiliensis*) over half a century ago, have reduced the extent of continuous forests (Struebig et al. 2008). In the present day, a significant expanse of approximately 60,551 hectares of unmodified forest within the Krau landscape is protected by the Department of Wildlife and National Parks Peninsular Malaysia (DWNP) as the Tengku Hasanal Wildlife Reserve (formerly known as Krau Wildlife Reserve (1965-2022)). In contrast, forest fragments outside the reserve are either protected as forest reserves by the Forestry Department of Peninsular Malaysia or private land.

Bat Sampling

The present study and Struebig et al. (2008) restricted bat sampling to the species-rich insectivorous bat assemblage. This group of bats is found in abundance in the forest understorey and is susceptible to habitat disturbances (Kingston 2013). We used four-bank harp traps (Francis, 1989) positioned across flight paths each night that followed trails, logging skids, streams, and swamp beds. Each harp trap was moved to a new position the following day. We identified the captured bats at the species level following Francis (2019). We recorded the forearm and weight of each bat, as well as their sex and reproductive status. We released the bats at the capture point within eight hours.

Community Matrix

All analyses were carried out in R version 2023.06.0+421 (R Core Team 2023). We calculated the sampling coverage to determine the sampling completeness of insectivorous bat assemblages at each site using the ‘iNEXT’ package (Hsieh et al. 2016). Using the *estimateD* function, we used a rarefied abundance-based approach based on Hill numbers (*q* = 2) to establish the 93% sampling coverage estimates for all sites. This is the highest value that can be used to ensure the extrapolation provides reliable estimates only up to approximately double the reference sample size in sites with low species richness and abundance. Using the reference sample size generated for each site based on the estimation of species diversity at the specified sampling coverage, we generated a community matrix by resampling species based on their occurrence probabilities, derived from their abundance at each site. In this community matrix, the columns represented sites, and the rows represented species. Using the full community matrix that included all bat species, we created submatrices considering only cave- and forest-roosting bats. See the supplementary data file Supplementary_Data_RawCommunityMatrix.csv for the raw community matrix.

Species Trait Data

We selected eight functional traits relating to the ability of species to fly in complex vegetation and acoustic detection and post-capture processing of insect prey. The traits include (a) body mass (g), (b) forearm length (mm), (c) wing area (m^2^), (d) wingspan (m), (e) maximum bite force (N), (f) echolocation call duration (ms), (g) echolocation call start frequency (kHz), and (h) echolocation call bandwidth (kHz) (Table S1). We measured body mass with a Pesola spring scale, and forearm length using a dial caliper. In addition, wing area is the surface area of both wings and the body, whereas wingspan was recorded as the distance between wingtips with wings fully extended. Maximum bite force was measured with a 500 N Kistler force transducer (Kistler Inc., Switzerland) connected to a Kistler charge amplifier (Kistler Inc., Switzerland) (see Senawi et al. 2015 for details).

Functional Metrics

A multidimensional functional space was built using Principal Component Analysis (PCA) using the *tr.cont.fspace* function in the ‘mFD’ package (Magneville et al. 2022). We identified that a two-dimensional functional trait space provided the most accurate representation based on the deviation between the trait-based distances and distances within the functional trait space. We used this space to compute the functional metrics using a community matrix of all insectivorous bats included in this study. This same process was repeated to assess functional diversity separately for cave-roosting and forest-roosting bats. We used the PC loadings to evaluate differences in species positions within the functional trait space. For all bats, variation along PC1 (68.5%) is primarily driven by size-related traits, while PC2 (19.7%) is mainly influenced by echolocation traits (Table S3). For cave-roosting bats, PC1 (74.5%) is largely explained by size and echolocation traits, with PC2 predominantly influenced by echolocation traits (19.8%) (Table S4). For forest-roosting bats, PC1 (72.4%) is primarily explained by size, while PC2 (15.5%) is mostly driven by echolocation traits (Table S5). A summary of the traits used in the analyses is provided in the supplementary data file Supplementary_Data_TraitSummary.csv.

Four functional diversity metrics were used in this study: 1) Functional richness (FRic) – FRic represents the area of multivariate functional space occupied by the focal assemblage as a proportion of that of the pooled assemblage; 2) Functional dispersion (FDis) – FDis measures the abundance-weighted mean distance of all taxa from the center of the functional space, which is the abundance-weighted mean trait value of the assemblage or community (Laliberté and Legendre 2010; Mouillot et al. 2013). Greater FDis indicates that trait combination within the community is more distinct and is more spread out from the centroid; 3) Mean Nearest Neighbor Distance (FNND) – FNND is the weighted distance to the nearest neighbor within the assemblage (Weiher et al. 1998). Lower FNND suggests co-existing species are spaced closer to one another within the functional trait space, reflecting a greater similarity in their traits; 4) Functional Identity (Fide) – Fide represents the average position of species along each axis of the functional trait space, based on the mean trait values weighted by abundance (Mouillot et al. 2013). Shifts in FIde may indicate changes in the mean trait values along the axes of the functional trait space of an assemblage.

Null Models

We conducted two separate null models to 1) investigate the effects of species loss due to fragmentation on the structure of functional trait space, and 2) identify the community processes driving disassembly in response to fragmentation. The null models were constructed using a custom function that incorporates ‘mFD’ package (Magneville et al. 2022) and ‘picante’ package in R (Kembel 2010).

To uncover the effects of species loss due to fragmentation on the structure of functional trait space, we generated 1000 random community matrices by sampling species at each value of species richness from the total species pool for the community matrix for all bat species, cave-roosting and forest-roosting bats using a custom function. The null distribution was generated using community matrices that were generated by randomizing species occurrences within the community matrix while maintaining the observed species richness at each site. This approach preserved the overall species richness gradient but removed the specific associations between species and sites. Following this, functional richness (FRic) and functional mean nearest neighbor distance (FNND) were calculated for each null community at each richness level. The values expected under null expectations were then compared to the observed values.

A separate null model approach was used to determine the community processes driving disassembly due to fragmentation. Each functional diversity metric was used in the randomization tests to determine if the observed functional diversity measures were different from those under null expectations based on 1000 simulations using the ‘independentswap’ method (Gotelli, 2000). This method maintains the species occurrence frequency and richness at each site. The observed values and the null expected values from the 1000 random assemblages for the full community matrix and the submatrices were used to generate the standardized effect size of each metric. The 95% confidence interval for each of the SES values was calculated. SES with a confidence limit greater or less than zero signifies that a particular metric deviates significantly, either higher or lower than random expectations.

Nestedness Analysis

We performed a nestedness analysis to determine the extent to which the assemblages exhibited nested patterns when fragments were ordered by species richness. Assemblages with lower species richness that are subsets of those with greater species richness indicate that species are lost following a deterministic pattern. Separate analyses were performed using presence-absence matrices for all bat species in the study, as well as submatrices specifically for cave-roosting and forest-roosting bats. Nestedness was quantified using the binary matrix. A series of 5,000 random community matrices was simulated for all the matrices, and the significance of the nestedness structure was evaluated with null model 3, which is the most conservative model with a smaller incidence of type I error (Rodríguez-Gironés and Santamaría, 2006). To assess whether the maximally nested matrices produced an ecologically meaningful nested arrangement to forest fragmentation, the order of forest fragments in the maximally nested matrix was correlated (Spearman correlation coefficient) with forest fragments ordered by fragment area (ha), the shortest Euclidean distance to the nearest unmodified forests (km), and distance to the nearest forest patch (km) (Struebig et al. 2008). An ecologically meaningful nested arrangement would produce a significant correlation (Rodríguez-Gironés and Santamaría, 2006).

Table S1. Mean measurements of functional traits governing the detection, prey pursuit, and processing of insectivorous bat species used in the present study. Sample sizes for all measurements included more than 5 individual bats for each species.

| **Species** | **Body Mass (g)** | **Forearm Length (mm)** | **Wing Area (m^2^)** | **Wingspan (m)** | **Maximum Bite Force (N)** | **Start Frequency (kHz)** | **Call Duration (ms)** | **Bandwidth (kHz)** |
| --- | --- | --- | --- | --- | --- | --- | --- | --- |
| **Cave Bats** |  |  |  |  |  |  |  |  |
| *Hipposideros bicolor* | 8.56 | 45.29 | 0.02 | 0.28 | 3.28 | 130.90 | 5.40 | 11.70 |
| *Hipposideros cervinus* | 10.20 | 49.55 | 0.02 | 0.30 | 4.20 | 139.90 | 4.10 | 25.70 |
| *Hipposideros diadema* | 46.20 | 84.39 | 0.04 | 0.50 | 27.71 | 61.60 | 12.90 | 8.70 |
| *Hipposideros kunzi* | 8.50 | 42.90 | 0.01 | 0.27 | 3.32 | 139.70 | 5.70 | 25.60 |
| *Hipposideros larvatus* | 17.40 | 56.50 | 0.02 | 0.34 | 9.96 | 104.30 | 5.00 | 16.80 |
| *Rhinolophus affinis* | 14.71 | 49.40 | 0.02 | 0.32 | 4.55 | 73.70 | 29.90 | 19.40 |
| *Rhinolophus refulgens* | 6.15 | 40.46 | 0.01 | 0.25 | 1.60 | 103.70 | 23.40 | 25.30 |
| *Rhinolophus stheno* | 8.54 | 45.61 | 0.01 | 0.29 | 2.64 | 86.10 | 27.50 | 24.70 |
|  |  |  |  |  |  |  |  |  |
| **Forest Specialists** |  |  |  |  |  |  |  |  |
| *Nycteris tragata* | 18.00 | 49.70 | 0.03 | 0.34 | 8.41 | 125.60 | 0.60 | 76.06 |
| *Hipposideros ridleyi* | 9.50 | 48.60 | 0.02 | 0.31 | 3.40 | 61.46 | 14.95 | 11.50 |
| *Rhinolophus luctus* | 29.00 | 63.00 | 0.03 | 0.41 | 17.9 | 42.00 | 64.30 | 3.06 |
| *Rhinolophus yonghoiseni* | 7.70 | 40.30 | 0.01 | 0.26 | 3.66 | 66.80 | 48.20 | 14.00 |
| *Rhinolophus trifoliatus* | 14.11 | 51.31 | 0.02 | 0.33 | 8.22 | 55.20 | 41.80 | 15.80 |
| *Kerivoula intermedia* | 3.30 | 29.07 | 0.01 | 0.22 | 0.80 | 189.43 | 1.70 | 94.41 |
| *Kerivoula papillosa* | 9.14 | 41.91 | 0.02 | 0.31 | 8.68 | 191.96 | 2.70 | 115.94 |
| *Kerivoula pellucida* | 4.42 | 30.98 | 0.01 | 0.24 | 1.81 | 226.07 | 2.40 | 155.62 |
| *Murina peninsularis* | 8.06 | 36.12 | 0.01 | 0.26 | 16.63 | 57.01 | 2.00 | 115.41 |
| *Murina suilla* | 4.07 | 29.43 | 0.01 | 0.21 | 4.33 | 164.24 | 2.00 | 94.01 |
| *Phoniscus atrox* | 5.20 | 33.80 | 0.01 | 0.25 | 2.88 | 169.24 | 2.80 | 92.48 |

Functional trait measurements were obtained from Kingston et al. (2000), Schmieder et al. (2012), Senawi et al. (2015), and Senawi and Kingston (2019). *Rhinolophus yonghoiseni* as *Rhinolophus sedulus*, and *Murina peninsularis* as *Murina cyclotis* in source literature*.* Bandwidth was measured by considering the full range of frequencies across all harmonics.

Table S2. Description of the continuous forests and fragments within the Krau landscape included in this study. The fragmentation variables for each site were obtained from Struebig et al. (2008). Fragment Area (ha) refers to Area, Euclidean Distance to the Nearest Continuous Forest (km) is denoted as Nearest Fragment, and Euclidean Distance to the Nearest Continuous Forest (km) is referred to as Isolation throughout the manuscript.

| **Site Name** | | **Coordinate** | **Site Category** | **Surrounding Land-Use** | **Fragment Area (ha)** | **Distance to the Nearest Forest Patch (km)** | **Euclidean Distance to the Nearest Continuous Forest (km)** |
| --- | --- | --- | --- | --- | --- | --- | --- |
| S01 | Kuala Lompat | 3°43′ N, 102°17′ E | CF | F | 137000 | - | - |
| S02 | Lubuk Baung | 3°43′ N, 102°13′ E | CF | F | 137000 | - | - |
| S03 | Kuala Serloh | 3°40′ N, 102°10′ E | CF | F | 137000 | - | - |
| S04 | Kuala Gandah | 3°36′ N, 102°09′ E | CF | F | 137000 | - | - |
| S05 | Jenderak Selatan | 3°38′ N, 102°17′ E | CF | F | 137000 | - | - |
| S06 | Perlok | 3°49′ N, 102°13′ E | CF | F, G | 137000 | - | - |
| F01 | Kemasul Temerloh | 3°23′ N, 102°11′ E | LF | A, O | 2883 | 0.6 | 7.7 |
| F02 | Kemasul Bentung | 3°26′ N, 102°08′ E | LF | A, O | 11339 | 1.2 | 7.5 |
| F03 | RTP Lembah Klau | 3°42′ N, 101°58′ E | SF | O, R | 100 | 2.3 | 7.4 |
| F04 | FELDA Jenderak | 3°37′ N, 102°19′ E | LF | O | 1838 | 1.9 | 2.5 |
| F05 | Bukit Besar | 3°22′ N,102°15′ E | MF | A | 551 | 1.2 | 18.1 |
| F06 | Klau Kecil | 3°47′ N, 101°53′ E | MF | R, O, G | 443 | 1.4 | 3.7 |
| F07 | Gunung Senyum | 3°41′ N, 102°27′ E | LF | O | 1356 | 0.6 | 12.3 |
| F08 | Paya Luas | 3°42′ N, 102°19′ E | MF | R, O, G | 353 | 1.0 | 2.1 |
| F09 | Paya Parit | 3°41′ N, 102°23′ E | SF | R, O, G | 31 | 0.6 | 5.0 |
| F10 | Hutan Kerdau | 3°39′ N, 102°25′ E | MF | R, O, G | 319 | 0.6 | 8.1 |
| F11 | Ulu Ragan | 3°36′ N, 102°20′ E | SF | O, R | 122 | 1.7 | 8.8 |
| F12 | Dato’ Shariff | 3°40′ N, 102°23′ E | MF | C, O | 161 | 0.4 | 6.9 |
| F13 | Kampung Gun | 3°33′ N, 101°58′ E | SF | O | 44 | 1.3 | 3.0 |
| F14 | Kampung Lebu | 3°38′ N, 101°56′ E | MF | O, G | 400 | 2.3 | 7.4 |
| F15 | Rumpun Makmur | 3°43′ N, 102°23′ E | MF | R, G | 160 | 1.8 | 4.6 |
| F16 | Tebing Tinggi | 3°51′ N, 102°23′ E | SF | C, G, R | 93 | 0.6 | 5.5 |
| F17 | Bukit Dinding | 3°49′ N, 102°24′ E | SF | R, O, G | 32 | 0.5 | 6.3 |
| F18 | Bukit Ketupat | 3°48′ N, 102°24′ E | SF | R, G | 115 | 0.3 | 6.6 |
| F19 | Paya Perak | 3°36′ N, 102°26′ E | SF | R, G | 100 | 0.7 | 13.4 |
| F20 | Batu Sawar | 3°39′ N, 102°28′ E | MF | O | 300 | 0.7 | 14.7 |
| F21 | Belungu | 3°44′ N, 102°33′ E | LF | O, R | 5225 | 1.8 | 11.0 |
| F22 | Desa Bakti | 3°48′ N, 102°28′ E | SF | A, P | 107 | 2.1 | 5.6 |
| F23 | Klau Besar | 3°75′ N, 101°89′ E | LF | O, R | 5581 | 0.7 | 5.5 |
| F24 | Jengka | 3°59′ N, 102°47′ E | LF | O, R | 2025 | 0.6 | 7.6 |
| F25 | Jambu Rias | 3°45′ N, 102°10′ E | SF | O, R | 32 | 1.2 | 4.6 |
| F26 | Karak | 3°41′ N, 102°05′ E | SF | O, R | 35 | 1.1 | 3.6 |

Land-use surrounding the site: A – *Acacia* plantation; C – cleared land; F – forest; G – mixed gardens; O – oil palm plantation; P – pine plantation; R – rubber plantation.

Table S3. Factor loading, the variance explained, and interpretation of each factor of the principal component analysis for all bats. Changes in species positions on PC1 are driven by traits related to size and PC2 is driven by echolocation traits.

| **Variables** | **Component** | |
| --- | --- | --- |
|  | **PC1** | **PC2** |
| Mean Body Mass (g) | 0.4081 | -0.1976 |
| Mean Forearm Length (mm) | 0.4153 | -0.0661 |
| Mean Wing Area (m^2^) | 0.3955 | -0.2681 |
| Mean Wingspan (m) | 0.4134 | -0.1529 |
| Mode Maximum Bite Force (N) | 0.3537 | -0.3044 |
| Call Duration (ms) | 0.2046 | 0.5779 |
| Start Frequency (kHz) | -0.3063 | -0.4333 |
| Bandwidth (kHz) | -0.2698 | -0.4969 |
|  |  |  |
| **Variance Explained** |  |  |
| Proportion of Variance | 68.5% | 19.7% |
| Cumulative Proportion | 0.6848 | 0.8816 |
| Interpretation | Size | Echolocation |

Table S4. Factor loading, the variance explained, and interpretation of each factor of the principal component analysis for cave-roosting bats. Changes in species positions on PC1 are driven by traits relating to size and bandwidth, and PC2 is driven by traits call duration and start frequency.

| **Variables** | **Component** | |
| --- | --- | --- |
|  | **PC1** | **PC2** |
| Mean Body Mass (g) | 0.4065 | -0.009 |
| Mean Forearm Length (mm) | 0.4048 | -0.06 |
| Mean Wing Area (m^2^) | 0.4074 | -0.04 |
| Mean Wingspan (m) | 0.405 | -0.0348 |
| Mode Maximum Bite Force (N) | 0.4034 | -0.0768 |
| Call Duration (ms) | -0.0257 | 0.79 |
| Start Frequency (kHz) | -0.2734 | -0.5827 |
| Bandwidth (kHz) | -0.3205 | 0.1546 |
|  |  |  |
| **Variance Explained** |  |  |
| Proportion of Variance | 74.5% | 19.8% |
| Cumulative Proportion | 0.7452 | 0.9428 |
| Interpretation | Size and echolocation | Echolocation |

Table S5. Factor loading, the variance explained, and interpretation of each factor of the principal component analysis for forest-roosting bats. Changes in species positions on PC1 are driven by size-related traits and PC2 is driven by bandwidth.

| **Variables** | **Component** | |
| --- | --- | --- |
|  | **PC1** | **PC2** |
| Mean Body Mass (g) | 0.3947 | -0.204 |
| Mean Forearm Length (mm) | 0.4054 | -0.0381 |
| Mean Wing Area (m^2^) | 0.3595 | -0.4021 |
| Mean Wingspan (m) | 0.3945 | -0.2117 |
| Mode Maximum Bite Force (N) | 0.2984 | -0.3531 |
| Call Duration (ms) | 0.3233 | 0.4197 |
| Start Frequency (kHz) | -0.3239 | -0.3711 |
| Bandwidth (kHz) | -0.3108 | -0.5584 |
|  |  |  |
| **Variance Explained** |  |  |
| Proportion of Variance | 72.4% | 15.5% |
| Cumulative Proportion | 0.7241 | 0.8794 |
| Interpretation | Size | Echolocation |

Table S6. Full performance of regression relationships between fragmentation measures and species richness and functional diversity metrics for all bats using Akaike’s information criterion corrected for small sample sizes (AICc). Candidate models for each response variable are listed in order from highest to lowest rank.

| **Response Variable** | **Model** | **AICc** | **Δ AICc** | **wAIC** |
| --- | --- | --- | --- | --- |
| Species Richness | Area | 134.43 | 0 | 0.27 |
|  | Area + Isolation | 134.96 | 0.53 | 0.21 |
|  | Isolation | 135.27 | 0.84 | 0.18 |
|  | Nearest Fragment | 136.29 | 1.86 | 0.11 |
|  | Area + Nearest Fragment | 136.52 | 2.09 | 0.09 |
|  | Area + Nearest Fragment + Isolation | 136.75 | 2.32 | 0.08 |
|  | Nearest Fragment + Isolation | 137.43 | 3 | 0.06 |
| Functional Richness (FRic) | Area + Isolation | -4.33 | 0 | 0.65 |
|  | Area + Nearest Fragment + Isolation | -1.38 | 2.95 | 0.15 |
|  | Isolation | -0.37 | 3.96 | 0.09 |
|  | Area | 0.49 | 4.82 | 0.06 |
|  | Nearest Fragment + Isolation | 2.43 | 6.76 | 0.02 |
|  | Area + Nearest Fragment | 3.27 | 7.61 | 0.01 |
|  | Nearest Fragment | 3.56 | 7.89 | 0.01 |
| Functional Dispersion (FDis) | Isolation | -32.6 | 0 | 0.27 |
|  | Nearest Fragment | -32.4 | 0.2 | 0.24 |
|  | Area | -32.33 | 0.27 | 0.24 |
|  | Area + Isolation | -30.2 | 2.41 | 0.08 |
|  | Nearest Fragment + Isolation | -30.19 | 2.41 | 0.08 |
|  | Area + Nearest Fragment | -29.88 | 2.72 | 0.07 |
|  | Area + Nearest Fragment + Isolation | -27.71 | 4.89 | 0.02 |
| Mean Nearest Neighbor Distance (FNND) | Isolation | -50.41 | 0 | 0.33 |
|  | Area + Isolation | -49.99 | 0.42 | 0.27 |
|  | Area | -49.18 | 1.23 | 0.18 |
|  | Nearest Fragment + Isolation | -47.85 | 2.55 | 0.09 |
|  | Area + Nearest Fragment + Isolation | -46.95 | 3.46 | 0.06 |
|  | Area + Nearest Fragment | -46.38 | 4.03 | 0.04 |
|  | Nearest Fragment | -45.64 | 4.77 | 0.03 |

Table S7. Full performance of regression relationships between fragmentation measures and species richness and functional diversity metrics for cave-roosting bats using Akaike’s information criterion corrected for small sample sizes (AICc). Candidate models for each response variable are listed in order from highest to lowest rank.

| **Response Variable** | **Model** | **AICc** | **Δ AICc** | **wAIC** |
| --- | --- | --- | --- | --- |
| Species Richness | Area | 82.8 | 0 | 0.33 |
|  | Nearest Fragment | 83.62 | 0.82 | 0.22 |
|  | Isolation | 83.73 | 0.93 | 0.2 |
|  | Area + Nearest Fragment | 85.3 | 2.5 | 0.09 |
|  | Area + Isolation | 85.52 | 2.72 | 0.08 |
|  | Nearest Fragment + Isolation | 86.26 | 3.57 | 0.05 |
|  | Area + Nearest Fragment + Isolation | 88.32 | 5.52 | 0.02 |
| Functional Richness (FRic) | Area | -29.79 | 0 | 0.34 |
|  | Area + Isolation | -29.3 | 0.49 | 0.27 |
|  | Area + Nearest Fragment + Isolation | -27.39 | 2.41 | 0.1 |
|  | Area + Nearest Fragment | -27.34 | 2.45 | 0.1 |
|  | Isolation | -27.29 | 2.5 | 0.1 |
|  | Nearest Fragment | -26.31 | 3.49 | 0.06 |
|  | Nearest Fragment + Isolation | -24.69 | 5.11 | 0.03 |
| Functional Dispersion (FDis) | Area | -21.8 | 0 | 0.37 |
|  | Nearest Fragment | -20.7 | 1.11 | 0.21 |
|  | Isolation | -20.26 | 1.55 | 0.17 |
|  | Area + Isolation | -19.74 | 3.07 | 0.08 |
|  | Area + Nearest Fragment | -19.05 | 2.76 | 0.09 |
|  | Nearest Fragment + Isolation | -17.68 | 4.13 | 0.05 |
|  | Area + Nearest Fragment + Isolation | -15.55 | 6.26 | 0.02 |
| Mean Nearest Neighbor Distance (FNND) | Isolation | -6.61 | 0 | 0.5 |
|  | Area | -3.83 | 2.77 | 0.12 |
|  | Nearest Fragment | -3.78 | 2.83 | 0.12 |
|  | Nearest Fragment + Isolation | -3.56 | 3.05 | 0.11 |
|  | Area + Isolation | -3.53 | 3.08 | 0.11 |
|  | Area + Nearest Fragment | -0.77 | 5.84 | 0.03 |
|  | Area + Nearest Fragment + Isolation | -0.07 | 6.53 | 0.02 |

Table S8. Full performance of regression relationships between fragmentation measures and species richness and functional diversity metrics for forest-roosting bats using Akaike’s information criterion corrected for small sample sizes (AICc). Candidate models for each response variable are listed in order from highest to lowest rank.

| **Response Variable** | **Model** | **AICc** | **Δ AICc** | **wAIC** |
| --- | --- | --- | --- | --- |
| Species Richness | Isolation | 70.73 | 0 | 0.32 |
|  | Area | 71.34 | 0.61 | 0.24 |
|  | Nearest Fragment | 71.49 | 0.76 | 0.22 |
|  | Area + Isolation | 73.46 | 2.73 | 0.08 |
|  | Nearest Fragment + Isolation | 73.61 | 2.88 | 0.08 |
|  | Area + Nearest Fragment | 74.31 | 3.58 | 0.05 |
|  | Area + Nearest Fragment + Isolation | 76.95 | 6.22 | 0.01 |
| Functional Richness (FRic) | Nearest Fragment | -15.23 | 0 | 0.39 |
|  | Area | -14.54 | 0.69 | 0.28 |
|  | Area + Nearest Fragment | -12.53 | 2.7 | 0.1 |
|  | Isolation | -12.09 | 3.13 | 0.08 |
|  | Nearest Fragment + Isolation | -11.98 | 3.25 | 0.08 |
|  | Area + Isolation | -11.38 | 3.85 | 0.06 |
|  | Area + Nearest Fragment + Isolation | -8.67 | 6.56 | 0.01 |
| Functional Dispersion (FDis) | Area | -39.25 | 0 | 0.35 |
|  | Isolation | -38.28 | 0.97 | 0.22 |
|  | Nearest Fragment | -38.24 | 1.01 | 0.21 |
|  | Area + Nearest Fragment | -36.67 | 2.58 | 0.1 |
|  | Area + Isolation | -35.85 | 3.4 | 0.06 |
|  | Nearest Fragment + Isolation | -34.87 | 4.38 | 0.04 |
|  | Area + Nearest Fragment + Isolation | -32.69 | 6.56 | 0.01 |
| Mean Nearest Neighbor Distance (FNND) | Nearest Fragment | -29.3 | 0 | 0.3 |
|  | Isolation | -29.18 | 0.11 | 0.28 |
|  | Area | -28.93 | 0.36 | 0.25 |
|  | Nearest Fragment + Isolation | -26.19 | 3.1 | 0.06 |
|  | Area + Isolation | -25.88 | 3.42 | 0.05 |
|  | Area + Nearest Fragment | -25.81 | 3.48 | 0.05 |
|  | Area + Nearest Fragment + Isolation | -22.08 | 7.22 | 0.01 |


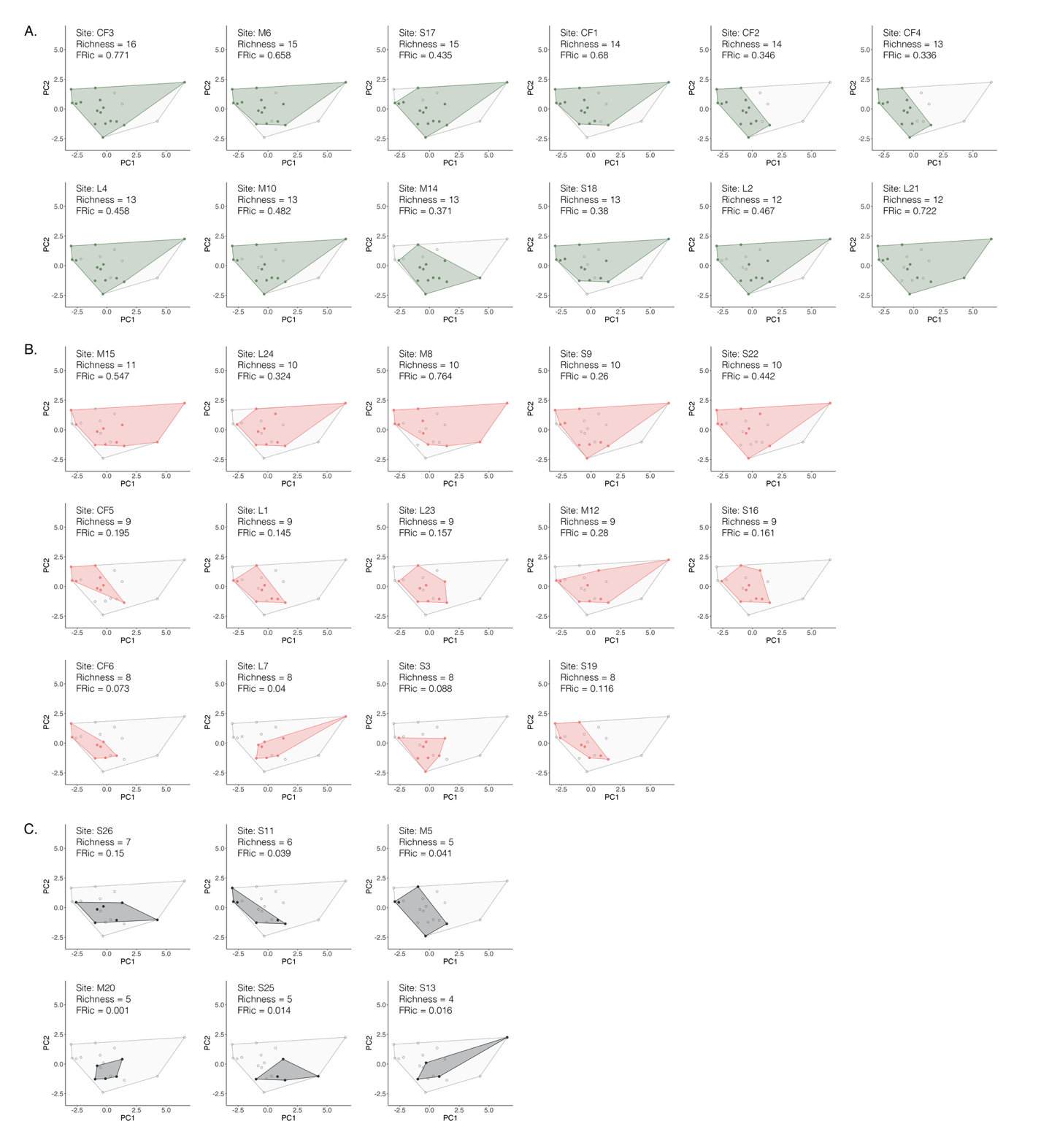


Figure S1. The loss of species at the periphery largely drives functional trait space contraction and reduces functional richness (FRic) across study sites. Panels showing (A) trait space before contraction phase, (B) trait space around the threshold of rapid contraction, and (C) trait space after contraction at reduced species richness. Each plot shows the convex hull for the site.

**References**

Struebig MJ, Kingston T, Zubaid A, Mohd-Adnan A, Rossiter SJ. 2008. Conservation value of forest fragments to Palaeotropical bats. Biological Conservation. 141: 2112-2126. <https://doi.org/10.1016/J.BIOCON.2008.06.009>

1. Francis CM. 1989. A comparison of mist nets and two designs of harp traps for capturing bats. Journal of Mammalogy. 70: 865-870. <https://doi.org/10.2307/1381730>

Francis CM. 2019. Field guide to the mammals of Southeast Asia second edition. Bloomsbury Wildlife.

1. Gotelli NJ. 2000. Null model analysis of species co-occurrence patterns. Ecology. 81: 2606-2621. <https://doi.org/10.2307/177478>

Hsieh TC, Ma KH, Chao A. 2016. iNEXT: An R package for rarefaction and extrapolation of species diversity (Hill numbers). Methods in Ecology and Evolution. 7: 1451-1456. <https://doi.org/10.1111/2041-210X.12613>

1. Kembel SW, Cowan PD, Helmus MR, Cornwell WK, Morlon H, Ackerly DD, Blomberg SP, Webb CO. 2010. Picante: R tool for integrating phylogenies and ecology. Bioinformatics. 26: 1463-1464. <https://doi.org/10.1093/bioinformatics/btq166>
2. Kingston T. 2013. Response of bat diversity to forest disturbance in Southeast Asia: insights from long-term research in Malaysia. pp. 169-185. In: Adam R, Pedersen SC (Eds.), Bat Evolution, Ecology and Conservation. Springer. New York.
3. Laliberté E, Legendre P. 2010. A distance-based framework for measuring functional diversity from multiple traits. Ecology. 91: 299-305. <https://doi.org/10.1890/08-2244.1>
4. Magneville C, Loiseau N, Albouy C, Casajus N, Claverie T, Escalas A, Leprieur F, Maire E, Mouillot D, Villéger S. 2022. mFD: an R package to compute and illustrate the multiple facets of functional diversity. Ecography. e05904. <https://doi.org/10.1111/ecog.05904>

Mouillot D, Graham NAJ, Villéger S, Mason NWH, Bellwood DR. 2013. A functional approach reveals community responses to disturbances. Trends in Ecology and Evolution. 28(3): 167-177. <https://doi.org/10.1016/j.tree.2012.10.004>

R Core Team. 2023. R: A language and environment for statistical computing. R Foundation for Statistical Computing. Vienna.

Rodríguez-Gironés MA, Santamaría L. 2006. A new algorithm to calculate the nestedness temperature of presence-absence matrices. Journal of Biogeography. 33: 924-935. <https://doi.org/10.1111/j.1365-2699.2006.01444.x>

Senawi J, Schmieder D, Siemers B, Kingston T. 2015. Beyond size – morphological predictors of bite force in a diverse insectivorous bat assemblage in Malaysia. Functional Ecology. 29: 1411-1420. <https://doi.org/10.1111/1365-2435.12447>

1. Weiher E, Clarke GDP, Keddy PA. 1998. Community assembly rules, morphological dispersion, and the coexistence of plant species. OIKOS. 81: 309-322. <https://doi.org/10.2307/3547051>
